# Quantified impacts of non‐pharmaceutical interventions on influenza circulation during the COVID‐19 pandemic in 13 African countries, 2020–2022

**DOI:** 10.1111/irv.13241

**Published:** 2024-01-18

**Authors:** Radhika Gharpure, Sonja J. Olsen, William W. Davis

**Affiliations:** ^1^ Influenza Division Centers for Disease Control and Prevention Atlanta Georgia USA; ^2^ Thailand MOPH‐U.S. CDC Collaboration Nonthaburi Thailand

**Keywords:** COVID‐19, nonpharmaceutical interventions, NPIs, seasonal influenza

## Abstract

Nonpharmaceutical interventions (NPIs) for SARS‐CoV‐2 disrupted circulation of influenza. We used data from 13 African countries and generalized linear models to identify associations between levels of NPIs, using the Oxford Stringency Index, and seasonal influenza activity, using parameters derived from 2020–2022 seasonal influenza surveillance. We found that for each step increase in school closings, the average percentage of respiratory specimens testing positive for influenza across the influenza season dropped by 20% (95% CI: 1–38%); no other NPI was significant. These findings may inform interventions to slow influenza circulation in pandemics and possibly during seasonal epidemics.

## 1 INTRODUCTION

During the recent COVID‐19 pandemic, nonpharmaceutical interventions (NPIs) were implemented globally to disrupt circulation of SARS‐CoV‐2. These NPIs also disrupted circulation of other respiratory viruses,^1^, ^2^, ^3^ including influenza virus.^4^ Because interventions were frequently rolled out in parallel, multiple studies have documented the layered effects of NPIs on influenza circulation during the COVID‐19 pandemic; however, it is important to characterize the effects of individual NPIs on influenza circulation, as these data can inform pandemic response plans. Two prior ecological studies found that school closings, limits on internal movements, restrictions on gatherings, and international border closures were each associated with reduced influenza circulation.^5^, ^6^ To broaden understanding of the effects of NPIs on influenza circulation, we repeated the methods from a previous Asian multi‐country study^5^ with data from African countries. This study aimed to determine if NPIs applied during the COVID‐19 pandemic reduced circulation of influenza, repeating methods from a previous Asian multi‐country study^5^ with data from African countries.

## 2 METHODS

We used methods previously described^5^ to analyze publicly available data from the WHO FluMart global repository for influenza data^7^ and the Oxford Stringency Index (OSI).^8^ Our first study used data from Asian countries; for this analysis, we sought a dataset from countries with similar tropical circulation patters and identified countries in Africa that met these inclusion criteria: (1) reported surveillance data to FluMart for ≥5 years during 2010–2019 in which ≥10 specimens were tested per week for ≥50% of the year (≥26 weeks); (2) reported OSI data on NPIs for COVID‐19 during 2020–2021; and (3) reported surveillance data to FluMart during 2020–2021 with ≥10 specimens tested per week for ≥26 weeks. Additionally, countries were included in 2022 analyses if they (1) reported OSI data on NPIs during 2022 and (2) reported data to FluMart during 2022 with ≥10 specimens tested per week for ≥26 weeks.

We downloaded data from FluMart* and from OSI†: the number of surveillance specimens tested per week for influenza, number testing positive for influenza viruses, influenza virus type, subtypes and lineages, and the OSI and 10 of its components: school closures (included all grade levels, although these were not specified), workplace closures, canceling public events, restrictions on gatherings, closing public transportation, stay‐at‐home requirements, restrictions on internal movement, international travel controls, public information campaigns, and mask mandates.

We constructed typical annual seasonal influenza epidemic curves (2010–2019, hereafter the “pre‐pandemic period”) for each country using the WHO R Shiny app,^9^ as previously described, which also determined if countries had one or two seasonal influenza peaks.^5^ Seasonal influenza data from January–June 2010 were excluded due to the 2009 influenza pandemic. Using threshold values from typical seasonal epidemic curves, we compared the start week, end week, and peak intensity week for each epidemic in the typical season (2010–2019) to seasonal epidemics occurring during March 2020–December 2022. We identified the start and end of an epidemic when the seasonal curve was, respectively, above or below the epidemic threshold for >2 continuous weeks. Additionally, we calculated the average percentage of respiratory specimens positive for influenza during 2010–2019 and compared with the percentage positive from 2020–2022 using Kruskal–Wallis tests.

We sought to identify associations between presence of 2020–2021 peaks or average percent positivity during a 2020–2021 season (or time period over a typical season if there was no 2020–2021 season) as dependent variables and the levels of one NPI or the OSI as an independent variable, controlling for population density. Separate models were run for each NPI. NPI variables were ordinal and reflected the intensity and geographic scope of implementation.‡ The number of peaks in the typical season across all countries (*n* = 16 per year) was the unit of analysis. For presence of an influenza season as the dependent variable, we used a generalized linear model with Poisson family, log link, and robust variance estimates to estimate the adjusted incidence rate ratio (IRR or relative risk) and 95% confidence intervals (95% CIs) of having a 2020–2021season. For average percent positivity across the season as the dependent variable, we used generalized linear models with logit link, binomial family, and robust option in STATA for error estimates. The exponentiated coefficients of the regression terms give the relative proportion ratio (RPR) or the ratio of the proportion of surveillance samples testing positive for influenza with an NPI intervention to the proportion of samples testing positive at baseline. We generated a correlation matrix with the STATA *correlate* command to examine relationships between NPI variables, with a value of ≥ ±0.8 indicating correlation. We conducted analyses with 2020–2021 data and 2020–2022 data. Analyses were performed in STATA 16/17.

## 3 RESULTS

Data from 13 countries met inclusion criteria and were included in the analysis: Cameroon, Côte d'Ivoire, Democratic Republic of the Congo, Kenya, Madagascar, Mali, Niger, Senegal, South Africa, Togo, Uganda, United Republic of Tanzania, and Zambia. Cameroon did not meet inclusion criteria for 2022. Across these 13 countries, the median and range of the number of weeks with zero specimens or no reporting to FluMart were 4 (0–22) in 2020, 3 in 2021 (1–14), and 1 in 2022 (1–16) (Figure S1).

**FIGURE 1:**
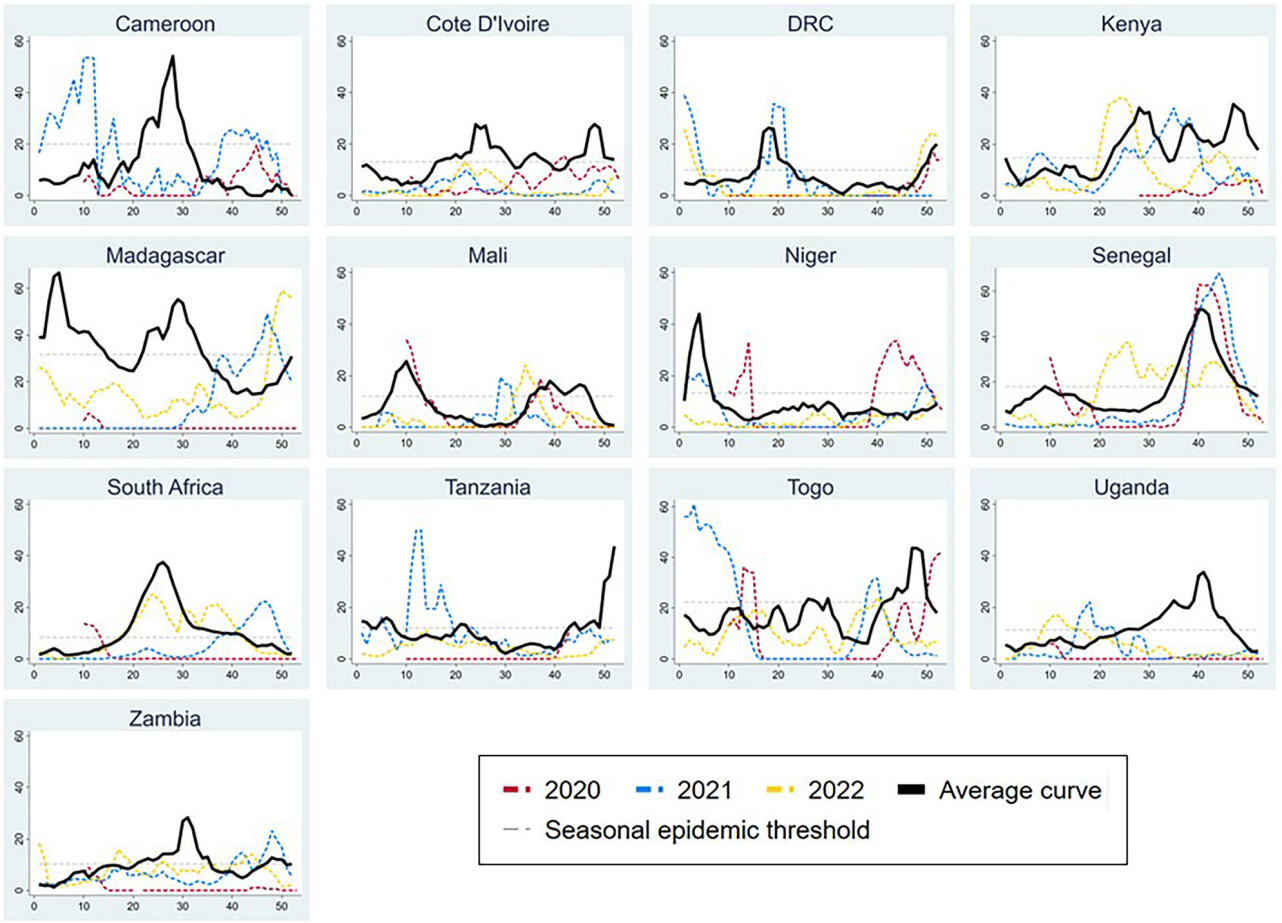
Seasonal (2020–2022) and average (2010–2019) influenza curves for 13 countries in Africa. Footnote: *x* axes depict epidemiological week (in increments of 10 weeks); *y* axes depict proportion positive for influenza (in increments of 20%). Average curves (2010–2019) generated using the WHO R Shiny app^9^; 2020 seasonal curves depicted as of Week 10, 2020 (declaration of COVID‐19 pandemic). Cameroon did not meet inclusion criteria for 2022.

Of 48 seasonal influenza epidemics expected in these 13 countries between Week 10, 2020 (declaration of COVID‐19 pandemic) and Week 52, 2022, only 26 (54%) occurred in 12 countries (Figure 1; expected seasonal peaks based on average curves are listed in Table S1). Côte d'Ivoire did not have any seasonal epidemics during 2020–2022. Of the 26 reported epidemics, 19 (73%) were shifted ≥4 weeks later from the average season; start dates ranged between −16 and 36 weeks from average. Season length was shorter than average for 17/26 (65%) epidemics. Atypical circulation continued through 2022 (Figure 1).

**FIGURE 2:**
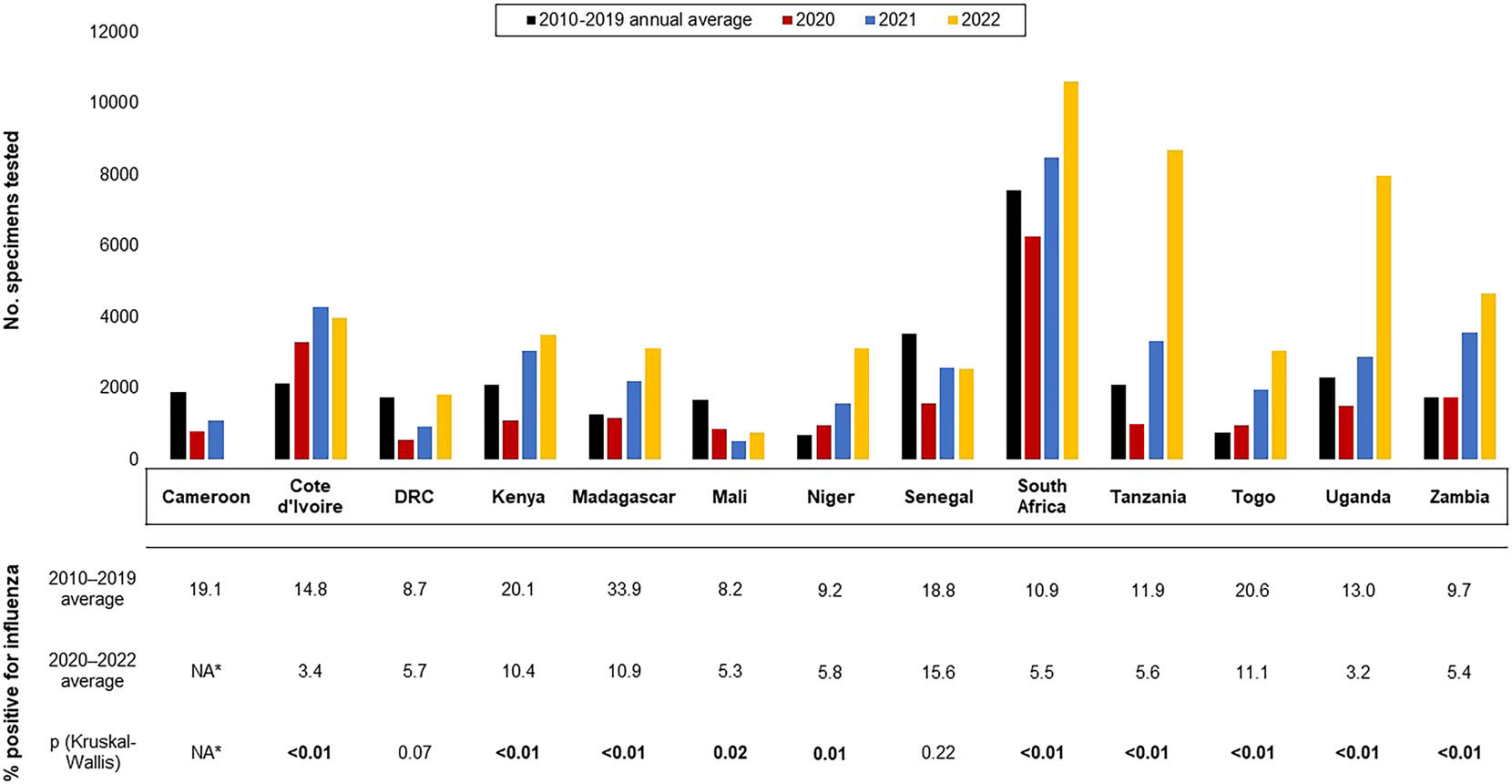
Number of surveillance specimens tested, by year, and average proportion positive for influenza for 13 countries in Africa, 2010–2019 and 2020–2022. Abbreviations: NA, not applicable. *Cameroon did not meet inclusion criteria for 2022.

During 2010–2019, the average number of specimens tested per year ranged from 709 to 7559 (Figure 2). This average dropped in 2020 (Weeks 10–52) for 9/13 (69%) countries, with a median decrease of 33% (interquartile range [IQR]: 0–52% decrease). The average number of specimens tested increased from the pre‐pandemic average for 9/13 (69%) countries in 2021 (median 47% increase; IQR: −27% to 102%) and 10/12 (83%) countries in 2022 (median 116% increase; IQR 31–262%). Most (83%) countries had a significantly lower proportion of surveillance respiratory specimens positive for influenza during 2020–2022 compared with the pre‐pandemic average; that is, circulation was much less during 2020–2022 than before the pandemic. Viruses detected during this time are in Table S2.

The average OSI composite score dropped from 2020 to 2022 as countries relaxed NPIs: 4 weeks before the typical season started (for all peaks in all countries), the average OSI was 51 (out of 100) in 2020, 38 in 2021, and 27 in 2022. Some NPIs such as school closings had even greater decreases in implementation; the average school closing score 4 weeks before the season started was 4.1 (out of 6) in 2020, 1.4 in 2021, and 0.3 in 2022.

Multivariable analysis for data from 2020–2021, controlling for population density, showed that school closings were significantly associated with reduced influenza positivity (Table 1). For each step increase in school closings, the average percent positivity across the influenza season dropped by 20% (95% CI: 1%–38%). We detected no other association between any NPI and the presence of an influenza season in 2020–2021 or in 2020–2022 (Tables S3–S5). The correlation matrix indicated that no two NPI variables were highly correlated (correlation ≥ ±0.8) (Table S6).

**TABLE 1:** Multivariable associations between nonpharmaceutical interventions^a^ and percent of respiratory specimens positive for influenza across 2020–2021 influenza epidemics^b^ for 13 countries in Africa.

Variable	Relative proportion ratio	95% CI
Oxford Stringency Index (OSI)^c^	0.99	0.97–1.01
School closings	0.80	0.64–1.00*
Workplace closures	0.96	0.78–1.19
Canceling public events	0.99	0.77–1.27
Restrictions on gatherings	0.93	0.82–1.06
Closing public transport	0.72	0.45–1.16
Stay at home orders	0.83	0.66–1.05
Restrictions on internal movements	0.86	0.65–1.12
International travel restrictions	0.96	0.66–1.40
Public information campaigns	1.01	0.73–1.40
Mask mandates	1.00	0.83–1.20

Abbreviation: CI, confidence interval.

^a^ OSI measures 4 weeks prior to the typical start of the influenza epidemic. All models controlled for population density.

^b^ For countries with no epidemic, dates for the typical epidemic season were used.

^c^ OSI is a composite measure of 23 individual COVID‐19 nonpharmaceutical interventions.

* Upper bound <1.00; statistically significant at *p* < 0.05.

## 4 DISCUSSION

Countries in this study experienced fewer and less intense seasonal influenza epidemics during 2020–2022 than in typical years. School closings were associated with a reduction in average percent positivity, that is, blunting of the influenza season, in 2020 and 2021. Multiple studies before the COVID‐19 pandemic documented the effects of school closings on seasonal and pandemic influenza,^10^ and prior literature suggests that the length and timing of the closure might impact its effectiveness.^11^

Two other studies also found that school closings were associated with reductions in seasonal influenza circulation during the COVID‐19 pandemic. Our study^5^ with data from Asian tropical countries used identical methods, and a study by Qiu et al.^6^ used data from temperate northern hemisphere countries to estimate effectiveness of individual NPIs on suppressing influenza circulation. Both studies identified additional NPIs associated with reductions in influenza circulation: restrictions on internal movements (both), restrictions on gatherings (both), and restrictions on international travel (Qiu). It is not clear why our analysis did not identify associations between these additional NPIs and influenza circulation. It is possible that adherence varied by the type of NPI (school closures, for example, may be practically easier to comply with or enforce than masking or travel restrictions) and cultural factors that might differ by country or region.^12^ We did not find associations between any NPIs and influenza circulation when we included 2022 data, possibly because overall levels of NPIs had dropped by about half since 2020 and schools were no longer being closed, but influenza circulation had not yet returned to pre‐pandemic seasonality patterns in 2022.^13^

Our study had several limitations. The ecological approach cannot allow for a causality assessment. A few countries had several weeks of missing data, especially in 2020 (*n* = 6 countries with >2 continuous weeks missing in 2020 and *n* = 2 in 2021), and it is possible that we missed some peaks during the epidemic in these data. Due to year‐round circulation of influenza and variable seasonal patterns in many tropical countries, it is challenging to establish seasonal epidemic curves and clearly define peaks and season start.^14^ Our use of average percent positivity across the season as a measure of circulation, however, is less dependent on peak height and season start and end dates. While the OSI metric quantifies government policies for NPIs, it does not account for the degree of compliance with these policies nor did it allow for a sub‐regional analysis within countries in our dataset.^8^ Although the OSI metrics account for regional application of NPIs, data from the countries in our dataset did not specify which region nor which types or grade levels of school were closed. An assessment of school closing by grade level (primary schools only, for example) might identify an even narrower intervention to reduce influenza circulation. The small sample size in this analysis (26 epidemics in 13 countries) limited additional analyses (e.g., effect modification of closing international borders) and quality of surveillance data was likely affected by the COVID‐19 pandemic, although we observed increased testing of specimens in 2021–2022, likely because of efforts to strengthen respiratory surveillance. Finally, we could find no way to control amount of time spent outdoors, a factor that could bias any NPI effect toward the null. Additional limitations are described in detail elsewhere.^5^

Our findings, that is, school closings during the COVID‐19 pandemic in selected African countries were associated with reduced influenza circulation, are consistent with findings from similar studies before and during the COVID‐19 pandemic. Repeated findings that preemptive school closures are associated with reduced seasonal influenza circulation suggest that schools may play a key role in influenza circulation, but additional studies are needed to better understand the effects of timing and duration of closures. Interventions to reduce transmission in schools during seasonal epidemics, such as vaccination, might also be explored. However, long, sustained school closures may not always be a practical option for seasonal influenza control or possibly even for pandemics due to severe and likely long‐lasting consequences on child health and development.^15^ Interventions to reduce transmission inside schools during seasonal epidemics and pandemics should be explored to leverage these findings for seasonal epidemic control and pandemic response.

## AUTHOR CONTRIBUTIONS

**Radhika Gharpure:** Data curation (equal); formal analysis (equal); investigation (equal); writing—original draft (equal); writing—review and editing (equal). **Sonja J. Olsen:** Conceptualization (lead); formal analysis (equal); investigation (lead); methodology (lead); supervision (lead); writing—original draft (supporting); writing—review and editing (equal). **William W. Davis:** Conceptualization (equal); data curation (equal); formal analysis (equal); investigation (lead); methodology (lead); writing—original draft (lead); writing—review and editing (equal).

## CONFLICT OF INTEREST STATEMENT

The authors have no conflict of interest to declare. The findings and conclusions in this report are those of the authors and do not necessarily represent the official position of the Centers for Disease Control and Prevention.

### PEER REVIEW

The peer review history for this article is available at https://www.webofscience.com/api/gateway/wos/peer-review/10.1111/irv.13241.

## Supporting information


**Figure S1.** Specimens tested by country and week.

**Table S1:** Parameters of typical and 2020–2022 influenza seasons, by country.

**Table S2:** Influenza viruses circulating in 13 countries in Africa, 2019–2022.

**Table S3:** Multivariable associations between nonpharmaceutical interventions* and presence (yes/no) of a seasonal influenza epidemic during January 2020–December 2021 for 13 countries in Africa.

**Table S4:** Multivariable associations between nonpharmaceutical interventions* and percent of respiratory specimens positive for influenza across 2020–2022 influenza epidemics** for 13 countries in Africa.

**Table S5:** Multivariable associations between nonpharmaceutical interventions* and presence (yes/no) of a seasonal influenza epidemic during January 2020–December 2022 for 13 countries in Africa.

**Table S6:** Correlation matrix* of OSI component variables, 2020–2022.

## Data Availability

Data used in this manuscript are available to the public on WHO FluMart and Oxford COVID‐19 Government Response Tracker websites.

## Footnotes

* Data from July 2010–December 2022; download date = July 24, 2023.

† Data from January 2020–December 2022; download date = June 15, 2023.

‡ For example, for school closings, 0 = no measures; 1 = recommendation to close or all schools open with alterations to normal operations in limited geographic areas; 2 = same as 1 but implemented nationally; 3 = require some school (e.g., only high school or only public school) closings in limited geographic areas; 4 = same as 3 but implemented nationally; 5 = requiring all levels of school closed in a limited geographic area; and 6 = same as 5 but implemented nationally.
