# Supplementary figures and images for: Quantified impacts of non‐pharmaceutical interventions on influenza circulation during the COVID‐19 pandemic in 13 African countries, 2020–2022
Source: Influenza Other Respir Viruses. 2024 Jan 18;18(1):e13241. doi: 10.1111/irv.13241 (PMC10796249; doi:10.1111/irv.13241)

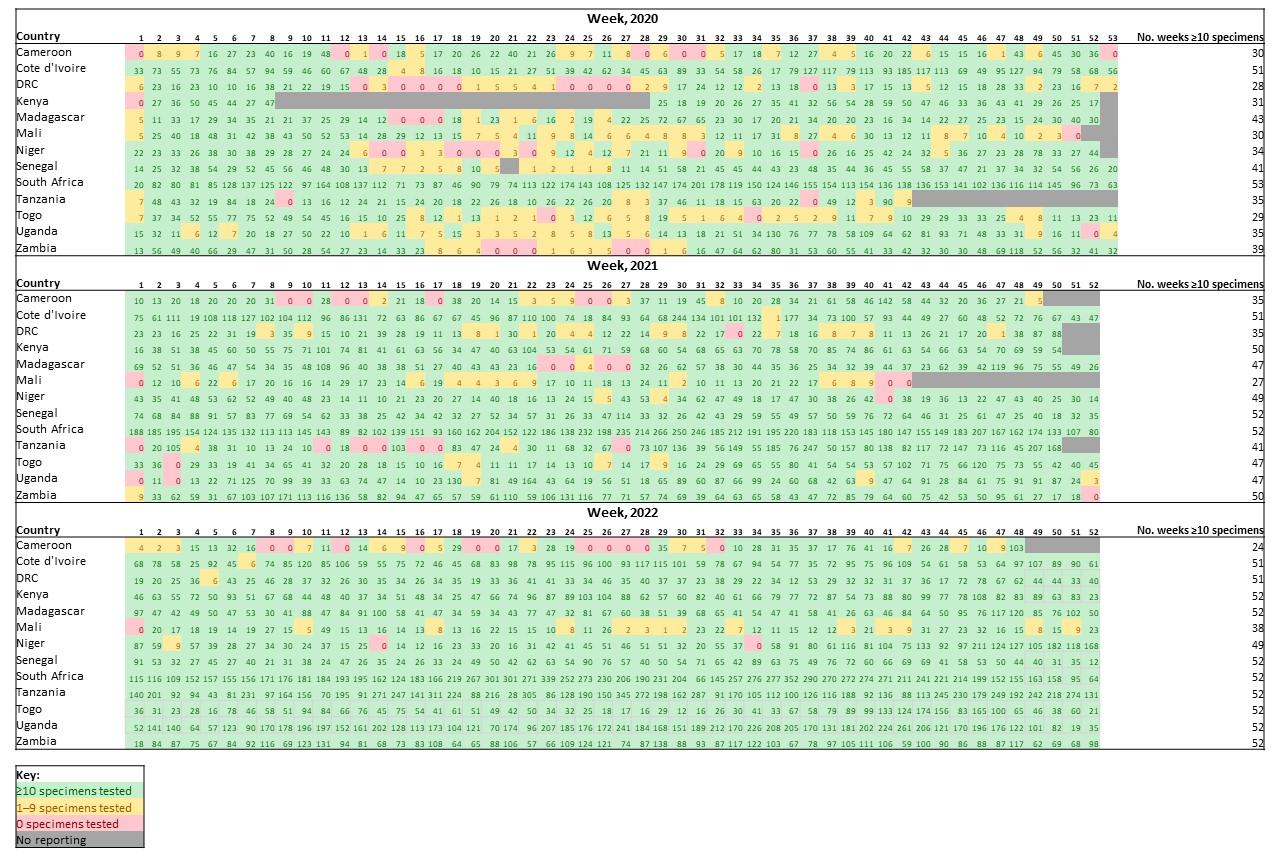

Supplement: Supplementary file 1 — Figure S1. Specimens tested by country and week. [file IRV-18-e13241-s006.jpg]
