# Supplementary material for: Quantified impacts of non‐pharmaceutical interventions on influenza circulation during the COVID‐19 pandemic in 13 African countries, 2020–2022
Source: Influenza Other Respir Viruses. 2024 Jan 18;18(1):e13241. doi: 10.1111/irv.13241 (PMC10796249; doi:10.1111/irv.13241)
Supplement: Supplementary file 2 — Table S1: Parameters of typical and 2020–2022 influenza seasons, by country. [file IRV-18-e13241-s004.docx]

**Supplemental Table 1: Parameters of typical and 2020–2022 influenza seasons, by country**

|  |  | **Start week** | | **Peak week** | | **End week** | | **Season length (weeks)** | | **Average percent positivity across season** | | | **Peak intensity (% positive)** | |
| --- | --- | --- | --- | --- | --- | --- | --- | --- | --- | --- | --- | --- | --- | --- |
| **Country** | **Year** | **Typical season** | **During pandemic** | **Typical season** | **During pandemic** | **Typical season** | **During pandemic** | **Typical season** | **During pandemic** | **Typical season** | **During pandemic** | **p (t-test)** | **Typical season** | **During pandemic** |
| Cameroon | 2020 | 22 | 2* | 28 | 11* | 32 | 17* | 10 | 15 | 15.3 | 6.0 | **0.02** | 54.1 | 53.6 |
| Cameroon | 2021 | 22 | 38 | 28 | 43 | 32 | 48 | 10 | 10 | 15.3 | 22.6 | **0.02** | 54.1 | 26.2 |
| Cote d'Ivoire | 2020 | 16 |  | 24 |  | 39 |  | 23 | 0 | 16.2 | 2.9 | **<0.01** | 27.7 | 0.0 |
| Cote d'Ivoire | 2020 | 43 |  | 48 |  | 52 |  | 9 | 0 | 18.0 | 9.8 | 0.07 | 27.8 | 0.0 |
| Cote d'Ivoire | 2021 | 16 |  | 24 |  | 39 |  | 23 | 0 | 16.2 | 3.2 | **<0.01** | 27.7 | 0.0 |
| Cote d'Ivoire | 2021 | 43 |  | 48 |  | 52 |  | 9 | 0 | 18.0 | 2.8 | **<0.01** | 27.8 | 0.0 |
| Cote d'Ivoire | 2022 | 16 |  | 24 |  | 39 |  | 23 | 0 | 16.2 | 4.6 | **<0.01** | 27.7 | 0.0 |
| Cote d'Ivoire | 2022 | 43 |  | 48 |  | 52 |  | 9 | 0 | 18.0 | 0.7 | **<0.01** | 27.8 | 0.0 |
| DRC | 2020 | 16 |  | 19 |  | 24 |  | 8 | 0 | 13.4 | 0.0 | **<0.01** | 26.3 | 0.0 |
| DRC | 2020 | 49 | 51 | 52 | 1* | 1 | 5* | 4 | 6 | 20.0 | 28.1 | 0.14 | 20.1 | 39.1 |
| DRC | 2021 | 16 | 16 | 19 | 19 | 24 | 22 | 8 | 6 | 13.4 | 24.8 | 0.82 | 26.3 | 35.9 |
| DRC | 2021 | 49 | 1* | 52 | 1* | 1 | 4* | 4 | 3 | 20.0 | 19.9 | 0.98 | 20.1 | 25.9 |
| DRC | 2022 | 16 |  | 19 |  | 24 |  | 8 | 0 | 13.4 | 0.0 | **<0.01** | 26.3 | 0.0 |
| Kenya | 2020 | 23 | 7* | 47 | 8* | 1* | 10* | 30 | 3 | 22.0 | 16.8 | 0.99 | 35.6 | 16.8 |
| Kenya | 2021 | 23 | 25 | 47 | 35 | 1* | 41 | 30 | 16 | 22.0 | 22.9 | 0.24 | 35.6 | 34.2 |
| Kenya | 2022 | 23 | 20 | 47 | 25 | 1* | 29 | 30 | 9 | 22.0 | 31.6 | **0.02** | 35.6 | 38.1 |
| Madagascar | 2020 | 22 |  | 29 |  | 34 |  | 12 | 0 | 37.4 | 0.0 | **<0.01** | 55.3 | 0.0 |
| Madagascar | 2021 | 1 |  | 5 |  | 15 |  | 14 | 0 | 40.1 | 0.0 | **<0.01** | 66.5 | 0.0 |
| Madagascar | 2021 | 22 | 45 | 29 | 47 | 34 | 50 | 12 | 5 | 37.4 | 40.0 | 0.59 | 55.3 | 49.1 |
| Madagascar | 2022 | 1 |  | 5 |  | 15 |  | 14 | 0 | 40.1 | 17.1 | **<0.01** | 66.5 | 0.0 |
| Madagascar | 2022 | 22 |  | 29 |  | 34 |  | 12 | 0 | 37.4 | 9.0 | **<0.01** | 55.3 | 0.0 |
| Mali | 2020 | 35 | 37 | 39 | 37 | 48 | 40 | 13 | 3 | 12.8 | 14.5 | 0.79 | 17.9 | 18.7 |
| Mali | 2021 | 7 |  | 10 |  | 14 |  | 7 | 0 | 12.3 | 0.0 | **<0.01** | 25.6 | 0.0 |
| Mali | 2021 | 35 | 29 | 39 | 29 | 48 | 32 | 13 | 3 | 12.8 | 16.7 | 0.60 | 17.9 | 19.4 |
| Mali | 2022 | 7 |  | 10 |  | 14 |  | 7 | 0 | 12.3 | 3.1 | **0.04** | 25.6 | 0.0 |
| Mali | 2022 | 35 | 32 | 39 | 34 | 48 | 37 | 13 | 5 | 12.8 | 18.1 | 0.20 | 17.9 | 24.2 |
| Niger | 2021 | 2 | 40** | 4 | 44** | 7 | 7 | 5 | 19 | 20.8 | 22.3 | 0.26 | 44.0 | 33.6 |
| Niger | 2022 | 2 |  | 4 |  | 7 |  | 5 | 0 | 20.8 | 2.2 | **0.01** | 44.0 | 0.0 |
| Senegal | 2020 | 34 | 38 | 41 | 42 | 49 | 48 | 15 | 10 | 35.0 | 48.8 | 0.05 | 52.0 | 63.0 |
| Senegal | 2021 | 34 | 38 | 41 | 44 | 49 | 50 | 15 | 12 | 35.0 | 48.4 | **0.04** | 52.0 | 68.1 |
| Senegal | 2022 | 34 | 20 | 41 | 26 | 49 | 48 | 15 | 28 | 35.0 | 25.9 | **0.05** | 52.0 | 37.4 |
| South Africa | 2020 | 18 |  | 26 |  | 43 |  | 25 | 0 | 19.8 | 0.0 | **<0.01** | 37.5 | 0.0 |
| South Africa | 2021 | 18 | 37 | 26 | 47 | 43 | 51 | 25 | 14 | 19.8 | 14.3 | 0.14 | 37.5 | 22.2 |
| South Africa | 2022 | 18 | 18 | 26 | 24 | 43 | 43 | 25 | 25 | 19.8 | 16.7 | 0.33 | 37.5 | 25.3 |
| Tanzania | 2020 | 43 | 11* | 52 | 13* | 8* | 20* | 17 | 9 | 16.7 | 27.2 | 0.11 | 43.7 | 50.0 |
| Tanzania | 2021 | 43 |  | 52 |  | 8* |  | 17 | 0 | 16.7 | 5.3 | **0.03** | 43.7 | 0.0 |
| Togo | 2020 | 42 | 50 | 48 | 3* | 51 | 13* | 9 | 15 | 34.5 | 44.7 | **0.03** | 43.7 | 60.9 |
| Togo | 2021 | 42 | 38 | 48 | 40 | 51 | 41 | 9 | 3 | 34.5 | 31.0 | 0.89 | 43.7 | 31.8 |
| Togo | 2022 | 42 |  | 48 |  | 51 |  | 9 | 0 | 34.5 | 6.8 | **<0.01** | 43.7 | 0.0 |
| Uganda | 2020 | 25 |  | 41 |  | 47 |  | 22 | 0 | 18.8 | 0.1 | **<0.01** | 33.6 | 0.0 |
| Uganda | 2021 | 25 | 16 | 41 | 18 | 47 | 24 | 22 | 8 | 18.8 | 15.8 | 0.81 | 33.6 | 22.2 |
| Uganda | 2022 | 25 | 9 | 41 | 11 | 47 | 17 | 22 | 8 | 18.8 | 14.1 | 0.69 | 33.6 | 17.2 |
| Zambia | 2020 | 21 |  | 31 |  | 36 |  | 15 | 0 | 11.3 | 0.0 | **<0.01** | 28.2 | 0.0 |
| Zambia | 2020 | 47 |  | 48 |  | 51 |  | 4 | 0 | 8.9 | 0.4 | **0.03** | 12.7 | 0.0 |
| Zambia | 2021 | 21 |  | 31 |  | 36 |  | 15 | 0 | 11.3 | 4.6 | **0.02** | 28.2 | 0.0 |
| Zambia | 2021 | 47 | 40 | 48 | 48 | 51 | 3* | 4 | 15 | 8.9 | 14.5 | 0.05 | 12.7 | 23.2 |
| Zambia | 2022 | 21 | 16 | 31 | 17 | 36 | 20 | 15 | 4 | 11.3 | 14.6 | 0.29 | 28.2 | 16.4 |
| Zambia | 2022 | 47 | 42 | 48 | 44 | 51 | 46 | 4 | 4 | 8.9 | 13.1 | 0.16 | 12.7 | 14.0 |

* Weeks corresponding to the following year.

** Weeks corresponding to the preceding year.

*** each row represents a typical seasonal peak identified using historical data

Peaks occurring during pandemic years were aligned to the closest average peak (before or after). Start week was defined as the first week of >2 continuous weeks above threshold; end week was the first week of >2 continuous weeks below threshold.

Excludes peaks beginning before week 10 for 2020 (Madagascar, Mali, Niger) and peaks extending to or past week 52 for 2022 (DRC, Madagascar, Tanzania).
