# Supplementary material for: Quantified impacts of non‐pharmaceutical interventions on influenza circulation during the COVID‐19 pandemic in 13 African countries, 2020–2022
Source: Influenza Other Respir Viruses. 2024 Jan 18;18(1):e13241. doi: 10.1111/irv.13241 (PMC10796249; doi:10.1111/irv.13241)
Supplement: Supplementary file 3 — Table S2: Influenza viruses circulating in 13 countries in Africa, 2019–2022. [file IRV-18-e13241-s001.docx]

**Supplemental Table 2: Influenza viruses circulating in 13 countries in Africa, 2019–2022**

|  | **Circulating influenza viruses just before COVID-19 pandemic (week 1 2019 to week 9 2020)** | | | | | **Circulating influenza viruses during the COVID-19 pandemic (week 10 - 52, 2020)** | | | | | **Circulating influenza viruses during the COVID-19 pandemic (Week 1 - 52, 2021)** | | | | | **Circulating influenza viruses during the COVID-19 pandemic (Week 1 - 52, 2022)** | | | | |
| --- | --- | --- | --- | --- | --- | --- | --- | --- | --- | --- | --- | --- | --- | --- | --- | --- | --- | --- | --- | --- |
| **Country** | % A (H1pdm09) | % A(H3) | % B Yam* | % B Vic | Total number of positive specimens | % A (H1pdm09) | % A (H3) | % B Yam* | % B Vic | Total number of positive specimens | % A (H1pdm09) | % A (H3) | % B Yam* | % B Vic | Total number of positive specimens | % A (H1pdm09) | % A (H3) | % B Yam* | % B Vic | Total number of positive specimens |
| Cameroon | 25.4 | 33.6 | 4.0 | 35.0 | 351 | 13.6 | 81.8 | 0.0 | 4.5 | 44 | 60.7 | 20.1 | 0.0 | 19.2 | 224 | NA** | NA** | NA** | NA** | NA** |
| Cote d'Ivoire | 12.6 | 64.8 | 2.1 | 20.1 | 483 | 5.5 | 53.7 | 0.0 | 40.8 | 201 | 69.7 | 28.4 | 0.9 | 0.9 | 109 | 0.8 | 48.3 | 0.0 | 50.8 | 118 |
| DRC | 30.2 | 20.9 | 3.5 | 37.2 | 86 | 10.0 | 70.0 | 0.0 | 0.0 | 10 | 0.0 | 48.9 | 0.0 | 24.4 | 45 | 28.8 | 20.5 | 0.0 | 50.7 | 73 |
| Kenya | 40.3 | 35.2 | 0.0 | 1.1 | 273 | 0.0 | 5.6 | 0.0 | 0.0 | 18 | 0.5 | 46.3 | 0.0 | 6.1 | 410 | 89.3 | 5.9 | 0.0 | 4.8 | 439 |
| Madagascar | 25.8 | 16.0 | 0.0 | 38.5 | 488 | 33.3 | 0.0 | 0.0 | 0.0 | 6 | 0.0 | 22.4 | 0.0 | 50.5 | 295 | 43.3 | 33.7 | 0.0 | 20.1 | 530 |
| Mali | 16.7 | 68.5 | 0.0 | 14.8 | 311 | 0.0 | 64.9 | 0.0 | 33.8 | 74 | 100.0 | 0.0 | 0.0 | 0.0 | 12 | 0.0 | 65.4 | 0.0 | 30.8 | 26 |
| Niger | 2.8 | 69.3 | 0.6 | 19.3 | 176 | 46.1 | 10.8 | 1.0 | 12.7 | 102 | 44.6 | 40.6 | 1.0 | 0.0 | 101 | 3.3 | 55.0 | 0.0 | 38.3 | 120 |
| Senegal | 0.9 | 65.1 | 2.1 | 31.1 | 585 | 0.4 | 45.1 | 0.0 | 50.8 | 264 | 89.5 | 3.6 | 0.0 | 0.6 | 334 | 1.4 | 71.4 | 0.0 | 27.2 | 497 |
| South Africa | 12.9 | 83.5 | 0.2 | 1.2 | 1252 | 87.0 | 0.0 | 0.0 | 8.7 | 69 | 51.8 | 16.0 | 0.0 | 24.7 | 413 | 34.7 | 30.9 | 0.0 | 28.7 | 1171 |
| Tanzania | 18.7 | 60.5 | *NS* | *NS* | 299 | 0.0 | 0.0 | *NS* | *NS* | 2 | 29.7 | 49.1 | 0.0 | 0.0 | 222 | 49.1 | 19.4 | 0.0 | 0.0 | 371 |
| Togo | 1.8 | 58.3 | *NS* | *NS* | 513 | 58.2 | 38.0 | *NS* | *NS* | 79 | 65.0 | 34.0 | 0.0 | 0.3 | 300 | 1.5 | 68.8 | 0.0 | 28.6 | 336 |
| Uganda | 1.8 | 58.3 | *NS* | *NS* | 184 | 37.5 | 50.0 | *NS* | *NS* | 8 | 1.0 | 93.2 | *NS* | 2.9 | 103 | 29.4 | 29.4 | *NS* | 40.7 | 418 |
| Zambia | 18.2 | 32.4 | 0.0 | 7.9 | 444 | 8.3 | 0.0 | 0.0 | 0.0 | 12 | 29.4 | 35.1 | 0.0 | 0.0 | 231 | 34.6 | 17.9 | 0.0 | 46.1 | 349 |

Abbreviations: NA, not applicable; Vic, Victoria; Yam, Yamagata.

*Two countries reported B/Yamagata in 2020 and 2021; however, the viruses identified have not been confirmed by a WHO Collaborating Center for influenza.

**Cameroon did not meet inclusion criteria for 2022.
