# Supplementary material for: Quantified impacts of non‐pharmaceutical interventions on influenza circulation during the COVID‐19 pandemic in 13 African countries, 2020–2022
Source: Influenza Other Respir Viruses. 2024 Jan 18;18(1):e13241. doi: 10.1111/irv.13241 (PMC10796249; doi:10.1111/irv.13241)
Supplement: Supplementary file 4 — Table S3: Multivariable associations between nonpharmaceutical interventions* and presence (yes/no) of a seasonal influenza epidemic during January 2020–December 2021 for 13 countries in Africa. [file IRV-18-e13241-s007.docx]

**Supplemental Table 3: Multivariable associations between nonpharmaceutical interventions* and presence (yes/no) of a seasonal influenza epidemic during January 2020–December 2021 for 13 countries in Africa**

| Variable | Incidence rate ratio | 95% CI |
| --- | --- | --- |
| Oxford Stringency Index (OSI)** | 1.00 | 0.99– 1.01 |
| School closings | 0.99 | 0.86–1.13 |
| Workplace closures | 1.08 | 0.92–1.26 |
| Cancelling public events | 1.06 | 0.88–1.28 |
| Restrictions on gatherings | 1.00 | 0.90–1.11 |
| Closing public transport | 1.08 | 0.83–1.40 |
| Stay at home orders | 0.94 | 0.80–1.11 |
| Restrictions on internal movements | 0.93 | 0.75–1.14 |
| International travel restrictions | 0.89 | 0.69–1.16 |
| Public information campaigns | 0.83 | 0.59–1.18 |
| Mask mandates | 1.03 | 0.88–1.20 |

Abbreviations: CI, confidence interval.

*OSI measures four weeks prior to the typical start of the influenza epidemic. All models controlled for population density.
**OSI is a composite measure of composite measure of 23 individual COVID-19 nonpharmaceutical interventions.
