# Supplementary material for: Quantified impacts of non‐pharmaceutical interventions on influenza circulation during the COVID‐19 pandemic in 13 African countries, 2020–2022
Source: Influenza Other Respir Viruses. 2024 Jan 18;18(1):e13241. doi: 10.1111/irv.13241 (PMC10796249; doi:10.1111/irv.13241)
Supplement: Supplementary file 5 — Table S4: Multivariable associations between nonpharmaceutical interventions* and percent of respiratory specimens positive for influenza across 2020–2022 influenza epidemics** for 13 countries in Africa. [file IRV-18-e13241-s005.docx]

**Supplemental Table 4: Multivariable associations between nonpharmaceutical interventions* and percent of respiratory specimens positive for influenza across 2020–2022 influenza epidemics** for 13 countries in Africa**

| Variable | Relative proportion ratio | 95% CI |
| --- | --- | --- |
| Oxford Stringency Index (OSI)*** | 0.99 | 0.98–1.01 |
| School closings | 0.86 | 0.71–1.04 |
| Workplace closures | 0.96 | 0.81–1.14 |
| Cancelling public events | 1.02 | 0.85–1.24 |
| Restrictions on gatherings | 0.98 | 0.88–1.09 |
| Closing public transport | 0.82 | 0.58–1.16 |
| Stay at home orders | 0.93 | 0.79–1.09 |
| Restrictions on internal movements | 0.88 | 0.68–1.13 |
| International travel restrictions | 1.00 | 0.71–1.42 |
| Public information campaigns | 1.03 | 0.78–1.36 |
| Mask mandates | 0.95 | 0.82–1.09 |

Abbreviations: CI, confidence interval.

*OSI measures four weeks prior to the typical start of the influenza epidemic. All models controlled for population density.
** For countries with no epidemic, dates for the typical epidemic season were used.
***OSI is a composite measure of composite measure of 23 individual COVID-19 nonpharmaceutical interventions.
