# Supplementary material for: Quantified impacts of non‐pharmaceutical interventions on influenza circulation during the COVID‐19 pandemic in 13 African countries, 2020–2022
Source: Influenza Other Respir Viruses. 2024 Jan 18;18(1):e13241. doi: 10.1111/irv.13241 (PMC10796249; doi:10.1111/irv.13241)
Supplement: Supplementary file 7 — Table S6: Correlation matrix* of OSI component variables, 2020–2022. [file IRV-18-e13241-s002.docx]

**Supplemental Table 6: Correlation matrix* of OSI component variables, 2020–2022**

|  | School closings | Workplace closures | Cancelling public events | Restrictions on gatherings | Closing public transport | Stay at home orders | Restrictions on internal movements | International travel restrictions | Public information campaigns | Mask mandates |
| --- | --- | --- | --- | --- | --- | --- | --- | --- | --- | --- |
| School closings | 1.00 |  |  |  |  |  |  |  |  |  |
| Workplace closures | 0.46 | 1.00 |  |  |  |  |  |  |  |  |
| Cancelling public events | 0.24 | 0.29 | 1.00 |  |  |  |  |  |  |  |
| Restrictions on gatherings | 0.41 | 0.50 | 0.65 | 1.00 |  |  |  |  |  |  |
| Closing public transport | 0.43 | 0.59 | 0.29 | 0.33 | 1.00 |  |  |  |  |  |
| Stay at home orders | 0.43 | 0.42 | 0.25 | 0.52 | 0.36 | 1.00 |  |  |  |  |
| Restrictions on internal movements | 0.54 | 0.30 | 0.36 | 0.38 | 0.45 | 0.47 | 1.00 |  |  |  |
| International travel restrictions | 0.76 | 0.21 | 0.18 | 0.21 | 0.33 | 0.35 | 0.54 | 1.00 |  |  |
| Public information campaigns | 0.29 | 0.11 | 0.09 | 0.06 | -0.06 | 0.12 | 0.09 | 0.27 | 1.00 |  |
| Mask mandates | -0.10 | 0.08 | -0.10 | 0.22 | 0.17 | 0.18 | 0.10 | 0.00 | 0.09 | 1.00 |
|  |  |  |  |  |  |  |  |  |  |  |

*A score of >0.80 indicates that variables are highly correlated.
